# Phosphorylation of kinase insert domain receptor by cyclin-dependent kinase 5 at serine 229 is associated with invasive behavior and poor prognosis in prolactin pituitary adenomas

**DOI:** 10.18632/oncotarget.10550

**Published:** 2016-07-12

**Authors:** Weiyan Xie, Chunhui Liu, Dan Wu, Zhenye Li, Chuzhong Li, Yazhuo Zhang

**Affiliations:** ^1^ Beijing Neurosurgical Institute, Capital Medical University, Beijing, China; ^2^ Beijing Tiantan Hospital, Capital Medical University, Beijing, China; ^3^ Neurological Department, Beijing Renhe Hospital, Beijing, China

**Keywords:** CDK5, KDR, phosphorylation, prolactinomas, invasiveness, Pathology Section

## Abstract

Pituitary adenomas constitute 15-20% of intracranial neoplasms. Previously we reported that cyclin-dependent kinase 5 (CDK5) is upregulated in pituitary tumors associated with activating protein p35, and plays an essential role in pituitary adenomas progression. Here we explored the mechanisms of CDK5 signaling in prolactin pituitary adenomas. Our data indicate that p35 expression and CDK5 activity are both significantly increased in human invasive prolactin pituitary adenomas as compared to noninvasive forms of pituitary adenomas. Inhibition of CDK5 activity suppressed cell migration and invasive ability in GH3 rat pituitary cells. We identified that CDK5 phosphorylates serine 229 residue (Ser-229) of kinase insert domain receptor (KDR), also known as VEGFR-2, in prolactin pituitary adenomas. Phosphorylation of Ser-229 is required for proper KDR surface localization. Phosphorylated Ser-229 in KDR (pSer-229) levels are significantly higher in noninvasive and invasive prolactin pituitary adenomas compared to normal pituitary tissues. In addition, our data indicated that higher KDR pSer-229 correlates with worse prognosis in patients with prolactin pituitary adenomas. In summary, our results illustrated that CDK5-mediated KDR phosphorylation controls prolactin pituitary adenoma progression and KDR pSer-229 serves as a potential prognostic biomarker for both noninvasive and invasive pituitary adenomas.

## INTRODUCTION

Pituitary adenomas are common neuroendocrine neoplasms arising from adenohypophyseal cells. Although pituitary tumors are typically benign, some forms are invasive and tend to recur. CDK5 is a member of the cyclin-dependent kinase (CDK) family, and its diverse function in regulating many signaling pathways are characterized in in the development and growth of the nervous system. For instance, CDK5 controls neuronal migration and differentiation [1]. Interestingly, CDK5 activity is required for cell invasion in prostate carcinoma, glioblastoma multiforme [2], pancreatic cancer [3], medullary thyroid cancer [4]. Inhibition of CDK5 activity decreases lung cancer cell motility [5]. Liu et al. have also reported that increased CDK5 expression in patients with non-small cell lung cancer is correlated with poor prognosis [6]. Previously, we have found that active CDK5 was present in normal human pituitary tissue, was associated with p35, and that CDK5 activity was upregulated in diverse types of pituitary adenomas [7], suggesting CDK5 activity may play a role in pituitary tumor progression. A better understanding of cellular and molecular mechanisms mediated by CDK5 may provide a therapeutic strategy for pituitary tumors.

Tumor angiogenesis is essential for tumor growth and invasion in pituitary adenomas [8, 9]. Complex vascularity correlates with increased size, advanced invasiveness, poor surgical outcomes, and malignancy of prolactin pituitary adenomas [10]. Vascular endothelial growth factor (VEGF) is critical for angiogenesis in pituitary adenomas and other neoplasms [11, 12]. Invasive pituitary prolactin adenomas exhibit an increase in vascularization than their noninvasive counterparts [13, 14]. Consistent with these findings, VEGF expression is significantly higher in invasive than noninvasive adenomas [15].

Kinase insert domain receptor (KDR), also known as Type 2 VEGF receptor (VEGFR2), mediates VEGF-induced angiogenesis under physiological and pathological conditions. KDR is a tyrosine kinase receptor, and triggers diverse intracellular responses, including the phosphoinositide 3-kinase (PI3K)/phosphorylation of protein kinase B (AKT) [16]. Abundant expression of VEGF and KDR has been identified in pituitary glands [17], and VEGF participates in the formation of vascular networks in pituitary tumors [18, 19]. Immunohistochemistry (IHC) has demonstrated that KDR is localized in vascular endothelial cells of normal and adenomatous pituitaries [10], suggesting that KDR may play a role in regulating angiogenesis. Generally, KDR signaling is initiated with tyrosine phosphorylation in the KDR cytoplasmic domain, leading to downstream effectors activation [20]. Phosphorylation at tyrosine-1212 is involved in autophosphorylation and kinase activation [21]. Mutation of both serine 1188 and serine 1191 impairs the ligand-dependent downregulation of KDR [22].

In the current study, we have sought to explore the biological functions of CDK5 and p35 in prolactin pituitary adenomas. We have found both p35 and p-CDK5 levels are significantly increased in invasive pituitary adenomas in comparison to noninvasive pituitary adenomas. We have identified KDR S229, located in the extracellular domain, as the only consensus phosphorylation site for CDK5. Both inhibition and depletion of CDK5 suppress cell migration, cell invasion and KDR pSer-229 level in pituitary cells. Surface expression of S229A-KDR (nonphosphorylatable) is dramatically lower than wide-type KDR (WT-KDR) in pituitary cell lines, suggesting that phosphorylation of Ser-229 (pSer-229) is required for KDR trafficking. KDR pSer-229 is upregulated in invasive pituitary adenomas and correlated with poor prognosis in patients with prolactin pituitary adenomas. Our results indicate that CDK5 mediates cell invasion through phosphorylation on KDR Ser-229 and KDR pSer-229 is a potential biomarker for pituitary adenomas.

## RESULTS

### Expression of CDK5 and p35 in prolactin pituitary adenomas

**Figure 1:**
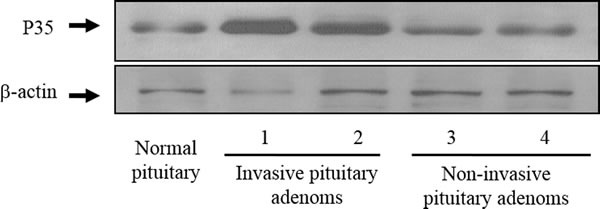
Representative Western blots of p35 in invasive and noninvasive prolactin pituitary adenomas Blots were re-probed with anti-β-actin antibody to ensure equal loading.

**Figure 2:**
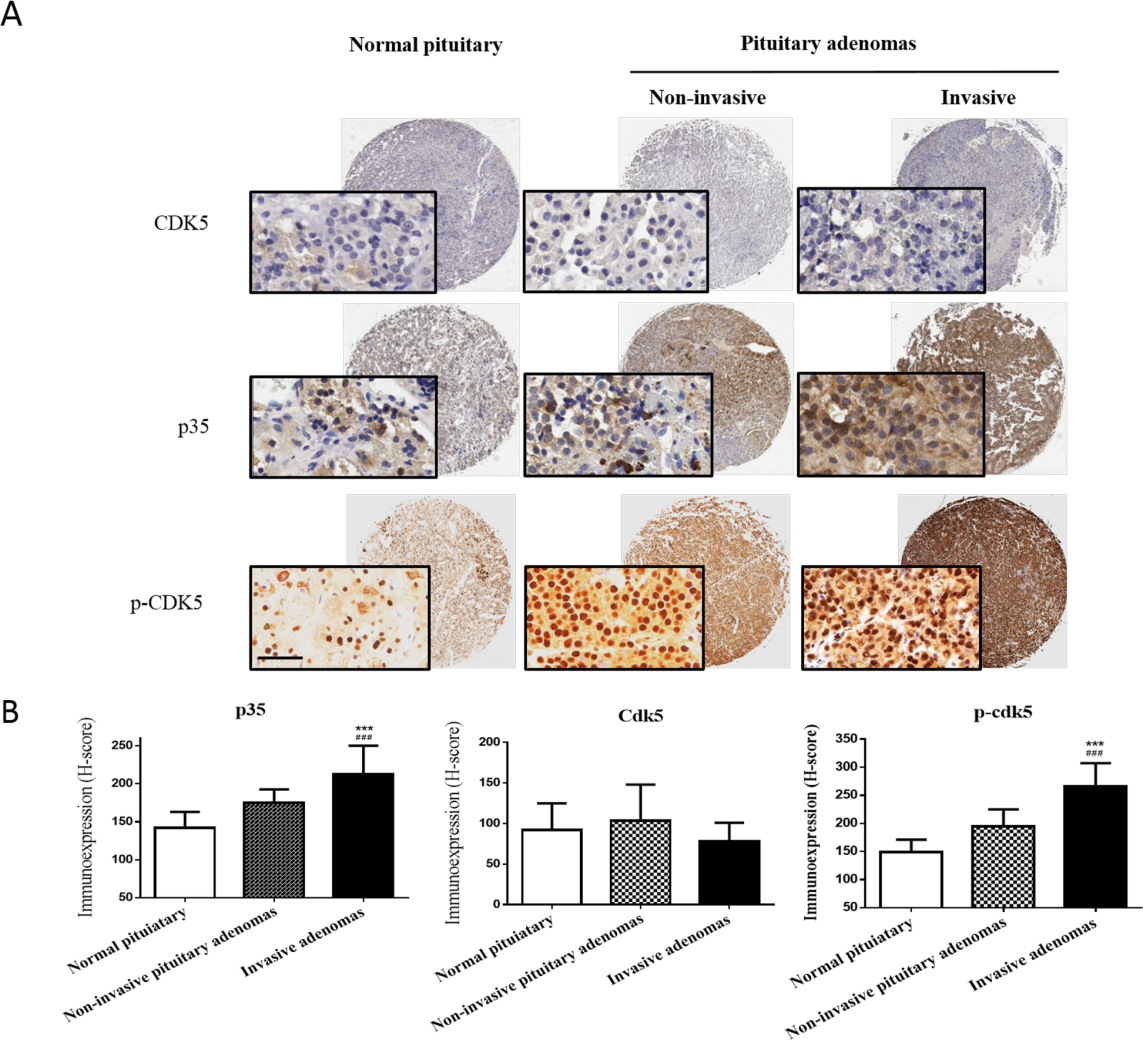
p35 protein and p-CDK5 levels are highly expressed in invasive pituitary adenoma tissue **A**. Representative images of CDK5, p35 and p-CDK5 staining of a tissue microarray showing normal pituitary tissue with low p35/p-CDK5 expression (left panel), noninvasive pituitary adenoma with moderate p35/p-CDK5 expression (middle panel), and invasive pituitary adenoma with high p35/p-CDK5 expression levels (right panel). No significant differences in CDK5 expression level were detected. The insets show 200× magnification of the low-power images (scale bar, 50 μm). **B**. Mean H-scores ± SD of CDK5, p35 and p-CDK5 staining of tissue microarrays. ***P<0.001 versus normal pituitary; ###P<0.001 versus noninvasive pituitary adenoma; ANOVA, followed by Newman–Keuls multiple comparison test.

First, we asked whether the expression of CDK5 or p35 differs in invasive and noninvasive prolactin pituitary adenomas. We found that p35 expression was markedly higher in invasive than in noninvasive prolactin pituitary adenomas (Figure 1). IHC staining confirmed the upregulation of p35 in invasive pituitary adenomas. Immunohistochemical expression of CDK5 and p35 is shown in Figure 2. CDK5/p35-positive staining was observed predominantly in the cytoplasm. Cytoplasmic p35 staining was significantly higher in noninvasive prolactin pituitary adenomas (mean H-score: 175) than in normal pituitary tissue (mean H-score: 142), with the highest expression observed in invasive prolactin pituitary adenomas (mean H-score: 212, Figure 2A, 2B), whereas no significant differences were detected in CDK5 H-scores.

To further examine CDK5 activity, we analyzed the phosphorylation levels of CDK5 in human prolactin pituitary adenomas. Expression of p-CDK5 was significantly higher in invasive (mean H-score: 266) than in noninvasive prolactin pituitary adenomas (mean H-score: 194). In addition, the p-CDK5 levels H-score was significantly higher in both invasive and noninvasive adenomas compared to normal pituitary tissues (mean H-score: 149, Figure 2A & 2B). These results indicated that CDK5 is specifically activated in human prolactin pituitary adenomas.

### CDK5 inhibition suppresses GH3 cells migration and invasion

**Figure 3:**
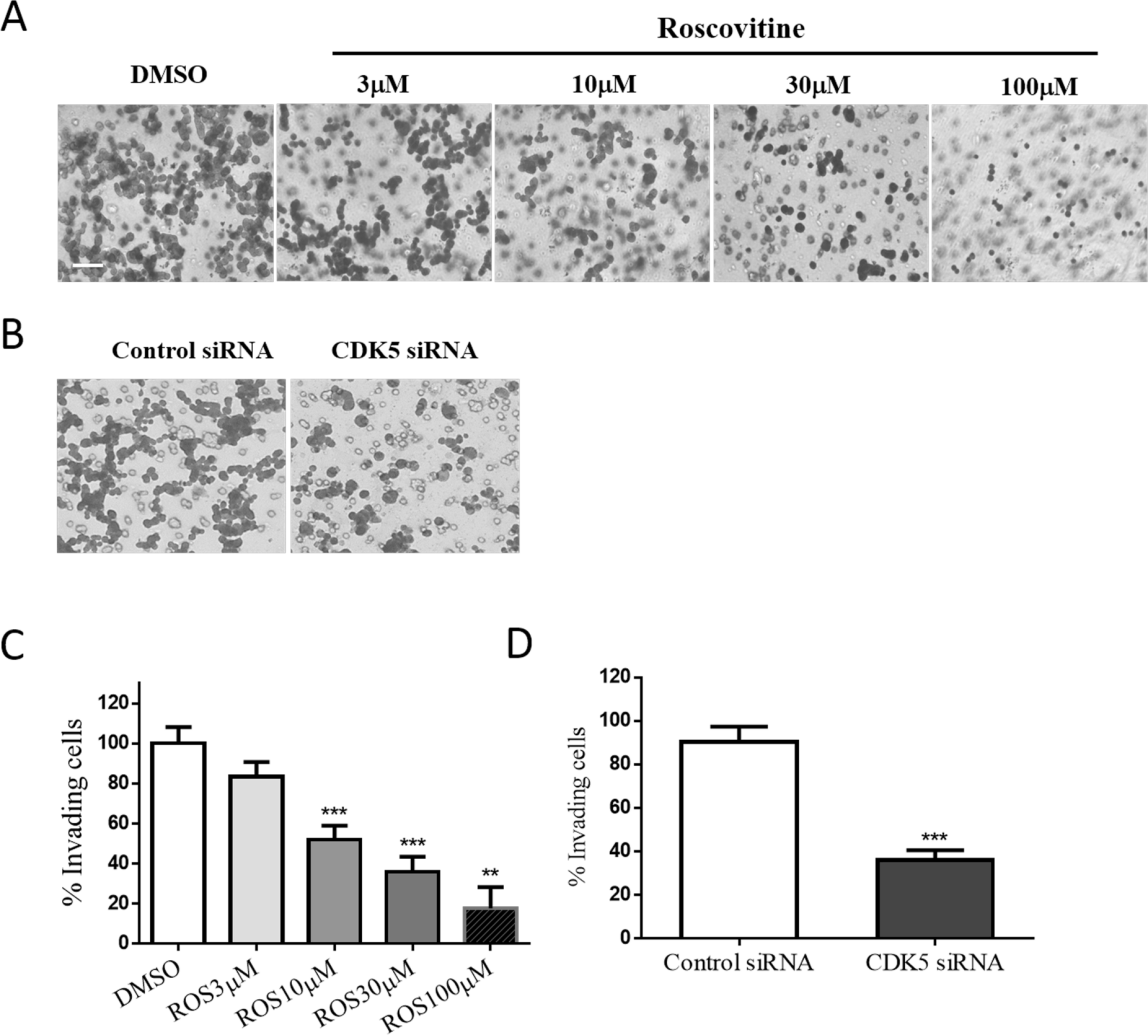

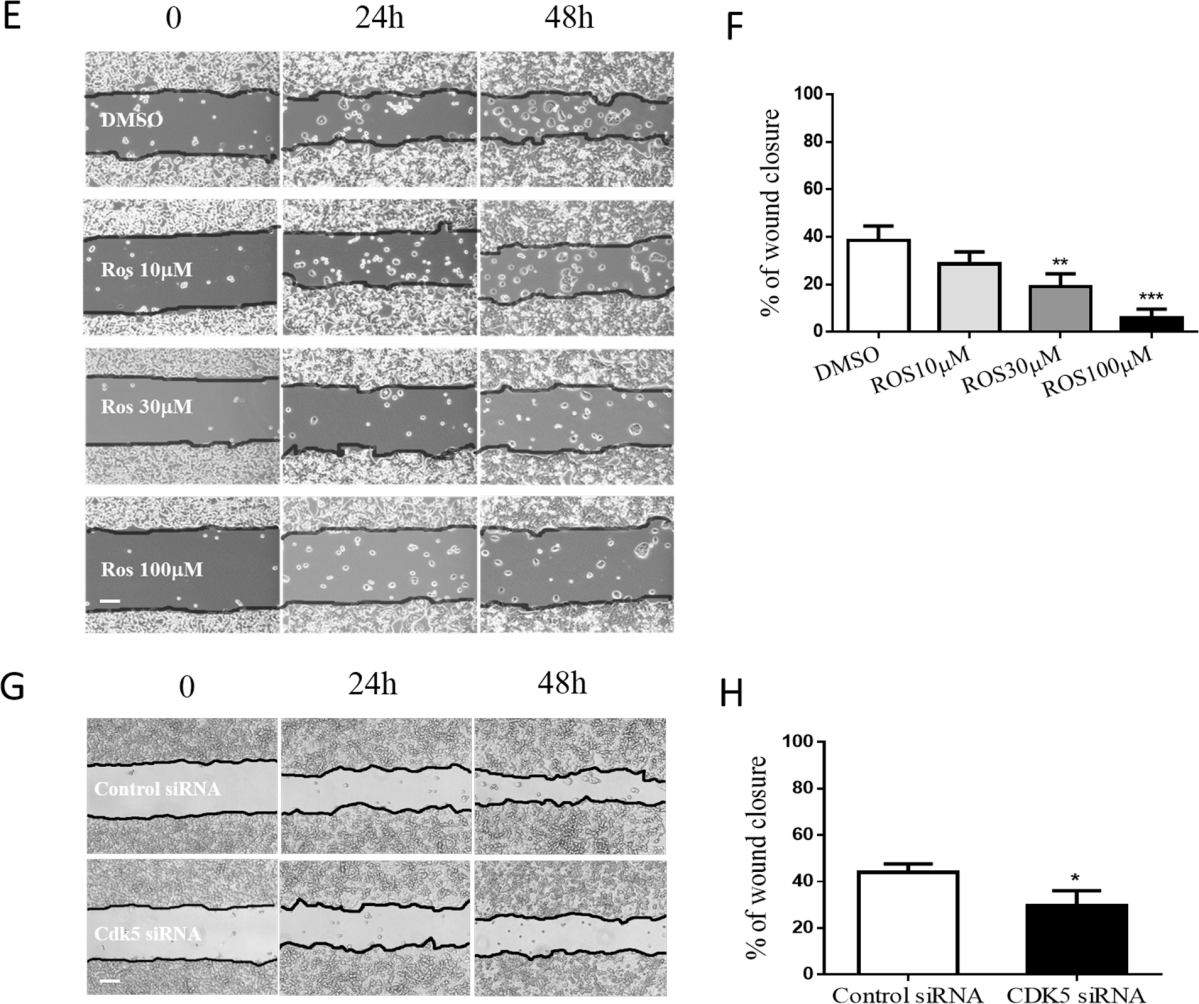
Both CDK5 inhibition and depletion affect the cell motility and cell migration activities of GH3 pituitary cells **A**. & **B**. GH3 pituitary cells were treated with roscovitine (0, 3, 10, 30, or 100μM) (A) or transfected with siRNAs as indicated (B) for 24 h and then migration was assayed in Transwell chambers (200× magnification, scale bar, 20μm). **C**. & **D**. Trend analysis of treatment dependent (C) or knock-down induced (D) invasiveness decrease of GH3 pituitary cells. **E**. & **G**. A scratch was applied to monolayers of GH3 cells that received roscovitine treatments at indicated concentrations (E) or siRNA transfections (G), cells were observed by phase-contrast microscopy (100×) and photographed at the indicated time points after wounding, scale bar, 200μm. **F** & **H**: Cell migration into the scratch wound after treatments (F) or knockdown (H) was quantified and presented as a percentage of wound healing (WH %) calculated by dividing migrated distance by scratched distance. *P<0.05, **P<0.01, ***P<0.001 versus control cultures.

To determine the influence of CDK5 on the migration and invasion of prolactin pituitary adenomas, we incubated GH3 rat pituitary cells with either treatments of roscovitine (a CDK5 inhibitor) in different concentrations. Involvement of endogenous CDK5 was also tested by using short interfering RNA (siRNA) targeting the CDK5 mRNA, knockdown efficiency was verified by western blot (data not shown). The migratory ability of the GH3 cells after 48 hours of treatment was determined by the Transwell migration assay. Our data indicate that migration of GH3 cells was significantly reduced by roscovitine treatment (Figure 3A & 3C) and CDK5 depletion (Figure 3B & 3D).

Wound-healing assays showed that the gap made in the confluent monolayer of GH3 cells was closed primarily by cell migration rather than cell proliferation. The results are also calculated and presented as percentage of wound closure as a function of time. At 48 hours, approximately 40% of the initial gap had closed in vehicle-treated cells, whereas 6 – 29% of the gap had closed in roscovitine (Figure 3E & 3F) or siRNA (Figure 3G & 3H) treated cells, indicating that roscovitine and CDK5 siRNA inhibited the cell motility. Taken together, these results suggested that roscovitine reduced gap closure by inhibiting cell motility. Our results show that cell migration and motility were significantly reduced by CDK5 knock-down. These results support the hypothesis that endogenous CDK5 plays a crucial role in GH3 cell migration and invasion.

### CDK5 associates and phosphorylates KDR-Ser229 in prolactin pituitary adenomas

**Figure 4:**
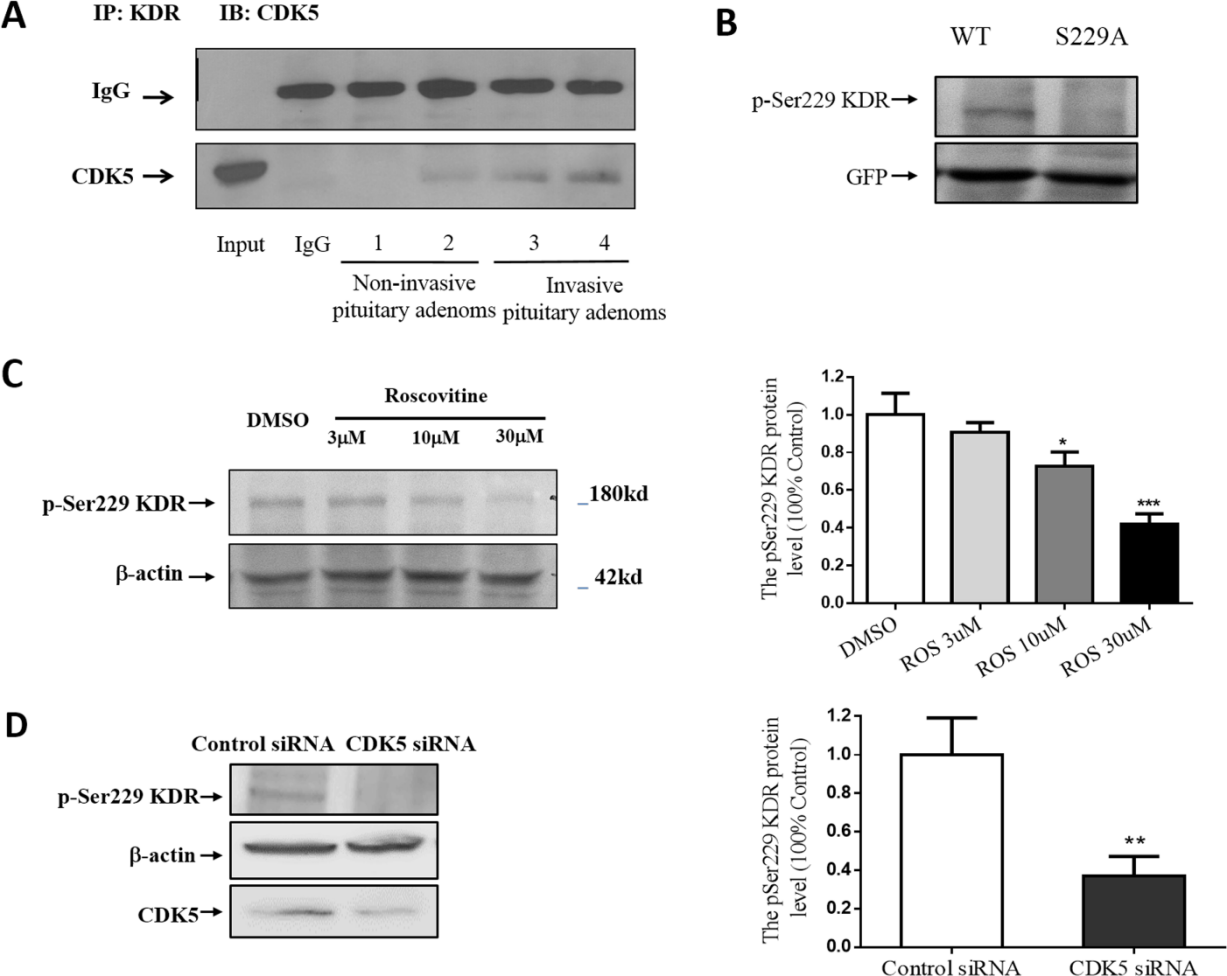

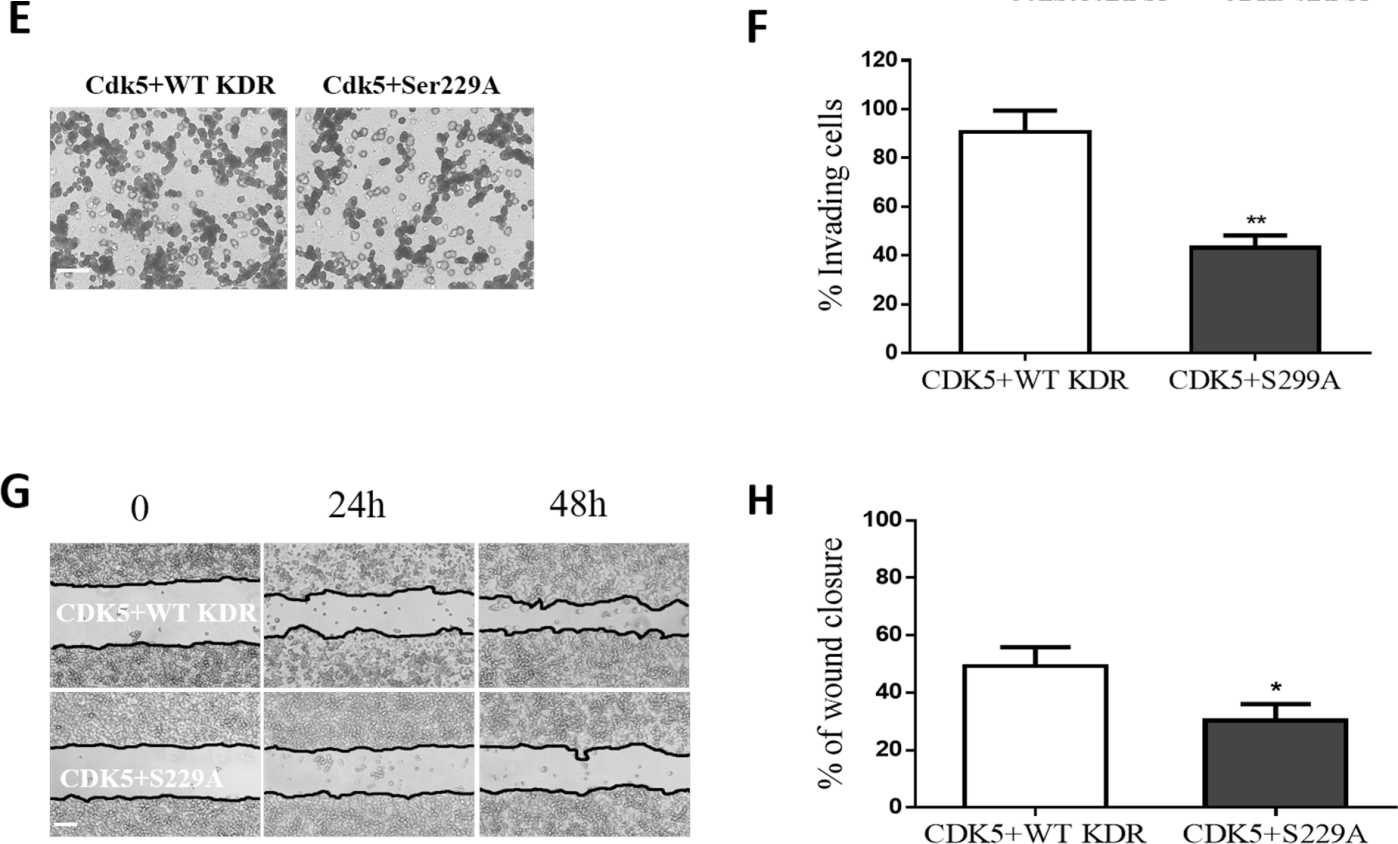
CDK5 regulates cell invasion via its phosphorylation of KDR at Ser-229 **A**. Interaction of CDK5 and KDR in prolactin pituitary adenomas. CDK5-specific antibody recognized the complex immunoprecipitated by KDR-specific antibody, but did not recognize normal IgG. **B**., GH3 pituitary cells were transfected with WT-KDR or S229A-KDR, and the blots were immunostained with site-specific pSer229 KDR antibody. Total cell lysates were immunoblotted with GFP and anti-actin as a loading control. **C**. Immunoblots (left) and densitometry analysis (right) showed KDR Ser-229 phosphorylation was reduced by roscovitine at indicated concentrations. β-actin served as a loading control. **D**. Immunoblots (left) and densitometry analysis (right) showed KDR Ser-229 phosphorylation was reduced by CDK5 siRNA at indicated concentrations. β-actin served as a loading control. **E**. Cell migration assay of GH3 pituitary cells cotransfected with CDK5 and WT-KDR/ S229A-KDR (200× magnification), scale bar, 20μm. **F**. Trend analysis of invasiveness in GH3 cells as indicated. **G**. Scratch wounds in monolayers of GH3 pituitary cell GH3 cells as indicated. Cells were observed by phase-contrast microscopy (100×), scale bar, 200μm. H: Cell migration into the scratch wound was quantified and presented as indicated. *P<0.05, **P<0.01, ***P<0.001 versus control cultures (two-tailed paired t test within each group. Error bars indicate SEM).

The pituitary contains abundant VEGF as well as KDR [23], and VEGF signaling participates in the neovascularization of pituitary tumors. Whether or not CDK5 affects the bioavailability or physiological function of KDR by phosphorylation has previously been unknown. To investigate this question, we examined whether inhibiting CDK5 activity has an effect on KDR function. To determine whether there is a direct interaction between CDK5 and KDR, we immunoprecipitated KDR from human pituitary adenoma lysates and probed with a CDK5-specific antibody. Immunoprecipitation of KDR led to coprecipitation of a detectable amount of CDK5 in noninvasive pituitary adenomas, with more intensive signaling in invasive pituitary adenomas (Figure 4A).

Phosphorylation of KDR is the key activation step for trafficking. CDK5 is a proline-directed serine/threonine kinase, and besides an absolute requirement for proline in the +1 position, CDK5 shows a marked preference for a basic residue in the +3 position. Its consensus phosphorylation sequence is (S/T) PX (K/H/R), where X can be any amino acid [24]. Using sequence analysis, we identified Ser-229 as the single putative CDK5 phosphorylation site in KDR (SPSH, located in extracellular Ig domain 3). To study Ser-229 activation, we raised a phosphospecific antibody against the pSer229 phosphopeptide. The pSer229-KDR phosphospecific antibody detected a ~250 kDa protein from wild-type green fluorescent protein (GFP)-tagged KDR expressed in GH3 cells, but it did not recognize GFP–KDR with a Ser (S) 229 to Ala (A) mutation (Figure 4B). The data indicated that pSer229-KDR phospho-antibody specifically recognized KDR phosphorylated at Ser229. To test whether CDK5 is able to phosphorylate KDR-Ser229 *in vivo*, we applied roscovitine (CDK5 inhibitor) and CDK5 siRNA to GH3 cells overexpressing GFP-tagged KDR. We found that both CDK5 inhibition and depletion significantly decreased the pSer229-KDR signal (Figure 4C & 4D).

To detect whether the inhibition of Ser229-KDR phosphorylation is required for CDK5-mediated migration and invasion, we cotransfected GH3 cells with CDK5 and WT-KDR or CDK5 with S229A-KDR (a nonphosphorylatable mutation). We found that migration (Figure 4E & 4F) and motility (Figure 4G & 4H) of GH3 cells were both significantly reduced in the S229A group. These data suggest that CDK5 mediates cell invasion and migration through KDR S229 phosphorylation.

### Ser229 is required for cell surface expression of KDR

**Figure 5:**
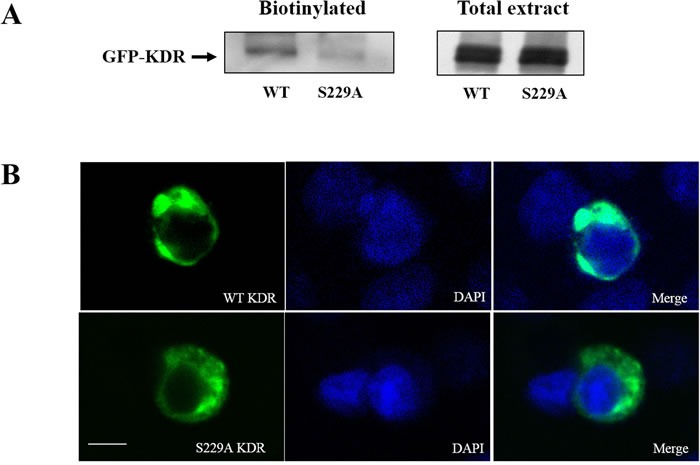
Ser-229 is necessary for cell surface expression of KDR **A**. Surface-biotinylated KDR shows that the expression of functional S229A-KDR at the cell surface was significantly lower than expression of WT-KDR in transfected GH3 pituitary cells. **B**. Differences in WT-KDR and S229A-KDR distribution in GH3 pituitary cells (630×, scale bar, 10 μm).

After confirming that CDK5 phosphorylates KDR at Ser229, we next investigated the function of S229A-KDR. We measured the total expression of WT-KDR and S229A-KDR in GH3 cells. GH3 cells were transfected with GFP-tagged WT-KDR or S229A-KDR. Probing the blots with an anti-GFP antibody detected an immunoreactive band of ~250 kDa in both WT and mutant KDR-expressing cells (Figure 5A), indicating that the S229A mutation did not affect the total expression of KDR. Next, we used a biotinylation assay to detect the cell surface expression of KDR in GH3 cells. As shown in Figure 5A, the expression of functional S229A-KDR was significantly lower compared to WT-KDR. To further validate this result, we transfected GH3 cells with S229A-KDR or WT-KDR. Confocal microscopy revealed that cells transfected with WT-KDR had greater cell surface expression of the receptor than cells containing S229A-KDR (Figure 5B). S229A-KDR staining was diffuse, without cell surface expression. These data suggest that CDK5 activity contributes to the distribution of functional KDR at the cell surface.

### Ser229-KDR phosphorylation in human prolactin adenoma tissue

**Figure 6:**
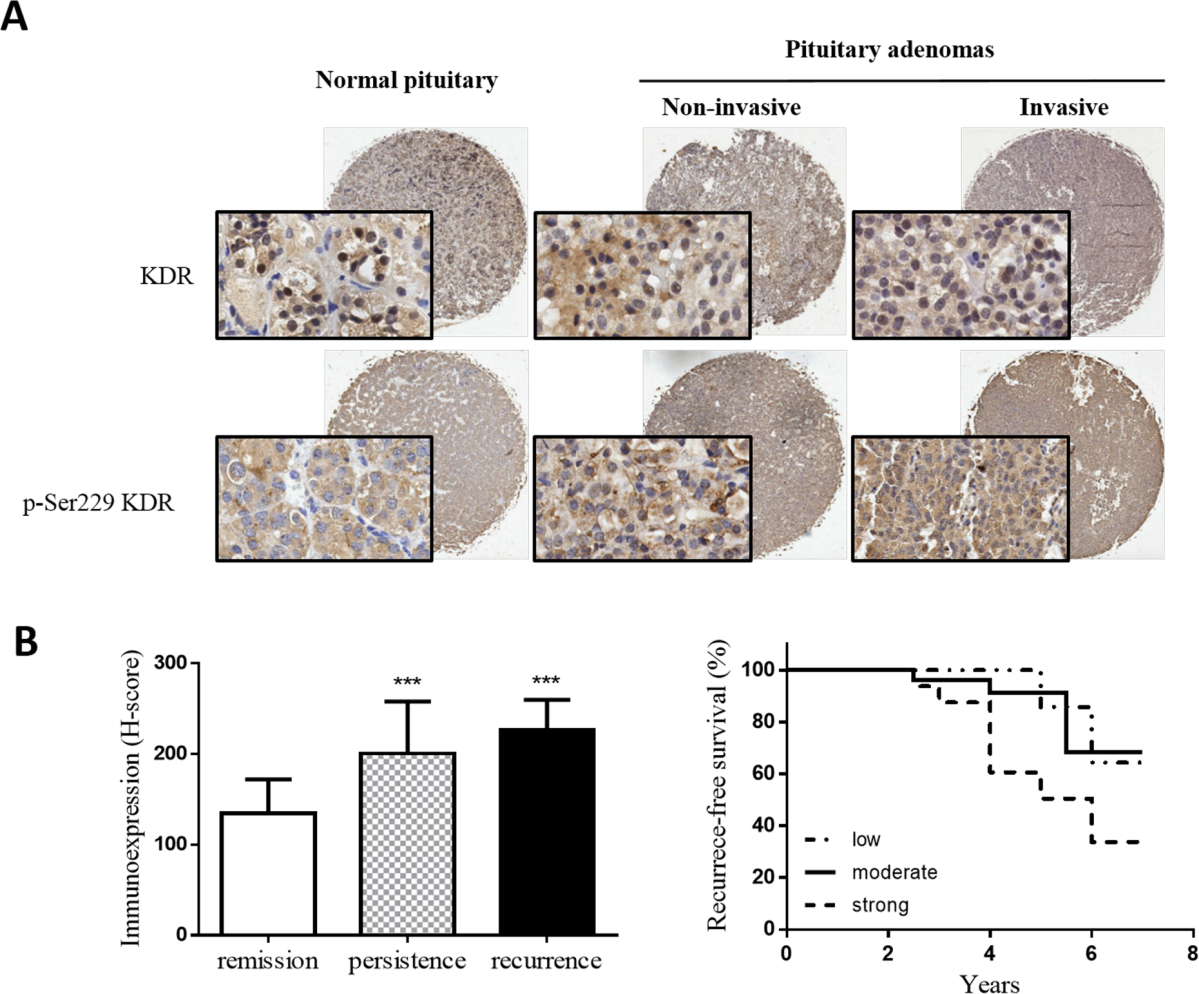
pSer229-KDR is highly expressed in invasive pituitary adenomas and associated with poor prognosis **A**. Representative images of pSer229-KDR staining of a tissue microarray, including low pSer229-KDR level in normal pituitary tissue (left panel), moderate pSer229-KDR level in noninvasive pituitary adenoma (middle panel) and high pSer229 KDR level in invasive pituitary adenoma (right panel). Insets show 200× magnifications of the low-power images, scale bar, 50μm. **B**. Mean H-scores of pSer229-KDR expression in patients in remission and patients with persistent or recurrent disease. ***P<0.001 versus patients in remission. (ANOVA, followed by Newman–Keuls test). C: Significant differences in recurrence-free survival according to pSer229-KDR expression status in 48 patients after surgical removal of prolactin pituitary adenomas. *P<0.05, by the log-rank test.

To investigate the clinical significance of Ser229-KDR phosphorylation, we carried out IHC staining of human prolactin adenoma tissue from a cohort of 48 patients with pSer229-KDR phosphoantibody. Expression of pSer229-KDR was significantly higher in invasive prolactin pituitary adenomas (mean H-score: 218) than in their noninvasive counterparts (mean H-score: 170). In addition, the pSer229-KDR H-score was significantly higher in both invasive and noninvasive adenomas than in normal pituitary tissue (mean H-score: 127) (Figure 6A). As shown in Figure 6B, the expression of pSer229-KDR was significantly higher in patients with disease recurrence or persistence than in patients in remission (P<0.001). The prognostic value of pSer229-KDR for recurrence-free survival in prolactin pituitary adenoma patients was evaluated by comparing the patients with weak, moderate, and strong pSer229-KDR expression. According to Kaplan-Meier survival analysis, patients with high pSer229-KDR expression had a distinctly shorter recurrence-free survival time than those with weak pSer229-KDR expression (P<0.05) (Figure 6C). These data suggest that pSer229-KDR might serve as a prognostic biomarker to predict the outcome of prolactin adenomas.

## DISCUSSION

In the present study, we have shown that p35 protein and p-CDK5 levels are significantly higher in invasive adenomas than in noninvasive adenomas. Inhibition and depletion of CDK5 activity suppresses cell migration and invasion of GH3 rat pituitary cells. KDR Ser-229 phosphorylation is required in CDK5 promoted cell invasion and this phosphorylation also contribute to the membrane trafficking of functional KDR to cell surfaces. Tissue microarray analysis of pSer229-KDR expression in 48 patients with prolactin pituitary adenomas indicated a significant correlation between high pSer229-KDR expression and poor prognosis.

Previously, CDK5 was reported to be uniquely involved in the development of the central nervous system and a key factor in neuronal migration. CDK5-dependent phosphorylation of the talin head domain at Ser425 prevents its ubiquitylation and degradation, regulating stability of cellular adhesion and cell migration [25]. Recently, CDK5 has been demonstrated to be involved in pancreatic [26], lung, and prostate [27] cancer. Although the *in vitro* and *in vivo* expression profiles of CDK5 have been investigated in several types of cancer, no published data on the expression and function of CDK5 in prolactin pituitary adenomas are available except for a report by Xie et al. [7]. The functional role of CDK5 activity in cell proliferation, migration, and invasiveness of pituitary adenoma cells remains to be elucidated. In our previous study [7], we found that active CDK5 was present in normal pituitary cells, associated with p35, and that CDK5 activity was upregulated in pituitary adenomas. CDK5 has also been shown to regulate angiogenesis and the migration of endothelial cells, and has been proposed as a target for antiangiogenic therapy [28]. Here, we have shown that both cell migration and invasion of pituitary cells were regulated by the CDK5 inhibitor roscovitine. At the concentrations applied in the current study, roscovitine would also inhibit CDK1 and CDK2; but CDK5 is the most likely target, because CDK2 expression is low in the anterior pituitary, and CDK1 is not expressed in corticotropes. By included CDK5 specific knock-down and nonphosphorylated KDR S229A mutant in this study, we confirmed endogenous CDK5 regulates cell migration and invasion through the phosphorylation on KDR S229.

Human prolactin pituitary adenomas vary greatly, ranging from small indolent tumors to large invasive ones. Invasive adenomas are less sensitive to dopamine agonists (DAs) than noninvasive tumors, and they are highly vascularized in general. KDR is the primary mediator of the mitogenic, angiogenic, and permeability-enhancing effects of VEGF, and KDR intracellular signaling is regulated by phosphorylation.

The role of CDK5 in GH3 cell migration and invasiveness could be attributable to its participation in decreased KDR cell surface bioavailability. Upon VEGF binding, VEGFR2 undergoes endocytosis, triggering downstream signaling cascades. Some pathways may reach full potential at the plasma membrane level, others are VEGFR2 subcellular localization dependent. For example, VEGFR2/ERK signaling is regulated by the trafficking speed of VEGFR2/NRP1 in the cytoplasm [29]. However, the mechanisms remain unknown. Dopamine agonists can normalize prolactin secretion in 80 to 90% of microadenoma patients and 70% of macroadenoma patients. CDK5 may phosphorylate the dopamine D2 receptor and attenuate downstream signaling [30]. The data suggest that CDK5 might also regulate DA resistance and tumor growth in prolactin pituitary adenomas. The CDK5 mechanisms involved in other subtypes of pituitary adenoma will be studied in the future.

Identification of new biomarkers that predict the prognosis and therapeutic resistance of prolactin pituitary adenomas is a priority. Here, we have demonstrated that CDK5 phosphorylates KDR at Ser-229 in prolactin pituitary adenomas, a step that is required for normal cell surface expression of KDR in cell migration and invasion *in vitro*. Therefore, KDR phosphorylation at Ser-229 may play a critical role in invasiveness and predicting poor prognosis of prolactin pituitary adenomas. In addition, our findings showed that CDK5 inhibitors can directly or indirectly block cell migration and invasion in prolactin pituitary adenomas.

## MATERIALS AND METHODS

### Patients

In the present study, we retrospectively reviewed 48 patients who had undergone pituitary surgery at Beijing Tiantan Hospital between 2008 and 2012. All patients had plasma prolactin (PRL) levels >200 ng/ml, and positive immunostaining for PRL. Patients with plurihormonal prolactin tumors were excluded from this study. Patients who were resistant to, or who could not tolerate dopamine agonist (DA) therapy, were included. Dopamine response was tested in 29 of the 48 patients; resistance to DA therapy was defined as previously described [31]. Medical therapy was interrupted at least 2 months before surgery. Tumor size was determined by MRI, and tumors were classified as microadenomas (<1 cm diameter), macroadenomas (>1 cm and <4 cm), and giant adenomas (>4 cm). The mean postoperative follow-up was 4.8 years (range: 2.5–7 years). Patients with no clinical or hormonal symptoms (PRL<30 ng/ml) and no radiological evidence of disease were considered to be in remission. Persistent disease was defined as a presence of increased plasma prolactin concentration with or without a mass visible by radiology. Tumor recurrence was defined as radiological evidence of tumor regrowth. Recurrence-free survival was measured from the date of surgery to the date of tumor recurrence. Patients were censored at the date of the last neuroimaging follow-up. Normal human anterior pituitaries of people who died of non-neurological or non-endocrine diseases were obtained from a donation program. Invasive pituitary adenomas were defined as Hardy–Wilson grade IV and/or Knosp grade III and IV [32, 33]. Twenty-three patients were diagnosed with invasive prolactin pituitary adenomas and 25 with noninvasive prolactin pituitary adenomas. The Ethics Committee of Beijing Tiantan Hospital study approved the protocol, and informed consent was obtained from all patients. The patient characteristics are summarized in Table 1.

**Table 1:** Clinical and pathological characteristics of the patients

Variables	Patients (*n*=48)
Sex, F/M	22/26
Age, years (mean ± SD, range)	39.3 ±10.7, 14-62
Macroadenoma (%)	23 (47.9%)
Microadenoma (%)	4 (8.3%)
Giant adenoma (%)	21 (43.8)
PRL (ng/ml) Grades 1a 1b 2a 2b	771.6 (209-5361) 23 2 20 3
Mean follow-up, years (mean ± SD, range)	4.8 ± 1.17, 2.5–7
Long-term follow-up results (%)	
Remission	18 (37.5%)
Persistence	16 (33.3%)
Recurrence	14 (29.2%)

F, female; M, male;

### Tumor samples and tissue microarray construction

Formalin-fixed paraffin-embedded tissue blocks were sectioned and stained with hematoxylin and eosin (H&E). Three 2.0 mm diameter core biopsies were selected from the paraffin-embedded tissue blocks and transferred to tissue microarrays (TMAs) using a Minicore Tissue Arrayer (Mitogen, UK). Tissue microarrays were cut into 4 μm sections using a serial microtome and samples were randomly ordered and anonymized on the TMA slides. To minimize loss of antigenicity, the microarray slides were processed within 1 week of cutting.

### IHC techniques and antibodies

In advance of IHC, TMA slides were stained with H&E and evaluated for quality and tumor content. TMAs were processed in a Leica BOND-III (Leica Biosystems, Germany) automated, random, and continuous-access slide staining system that simultaneously performed several IHC assays. A Bond Polymer Refine Detection system (Leica Biosystems, Germany) was used for detection of primary antibodies. Appropriate positive and negative controls were used for each antibody, and TMAs were stained for each antibody in the same run to avoid interassay variability. The immunostained slides were examined for expression using an Aperio AT2 digital scanner (Leica Biosystems, Germany). Primary antibodies anti-KDR (ab2349, 1/300), anti-p35 (ab66064, 1/100), and anti-CDK5 (ab40773, 1/200) were obtained from Abcam (Cambridge, MA, USA). Anti-p-CDK5 (Sc-12918, 1/100) was sourced from Santa Cruz Biotechnology (Dallas, TX, USA). Anti-pSer229-KDR (4 μg/ml, Abmart), was commercially developed using standard methods by injection of specific KDR-phosphothreonine peptide Ac-VVL(pS)PSHGIE-amide into mice at the Abmart antibody production facility, Shanghai, China. The optimal titer of primary antibodies had been determined in previous experiments. The percentage of immunostaining and the staining intensity (0, negative; 1+, weak; 2+, moderate; and 3+, strong) were recorded and an H-score was calculated as follows:

H-score = (% cells 1+) + 2(% cells 2+) + 3(% cells 3+).

The maximum H-score was 300, corresponding to 100% of cells stained with strong intensity. Based on the H-score, pSer229 KDR staining in the tissue sections was categorized as weak (H-score of ≤100), moderate (100<H-score≤200), or strong (H-score >200).

### Cell culture

Rat pituitary cells (GH3) were originally obtained from the China Infrastructure of Cell Line Resources (Beijing, China) and cultured at 37°C in 35 mm dishes in a humidified atmosphere of 95% air and 5% CO_2_. The culture medium was Dulbecco's minimum essential medium (DMEM) with 10% fetal bovine serum (FBS). Cultures were fed every other day. The cell lines were also genotyped to rule out cross-contamination and their morphology was regularly examined.

### Plasmid construction and KDR inhibitor

The pLenti-C-mGFP–KDR (RG219851) and CDK5 siRNA (SR507441) construct were purchased from OriGene Technologies (Rockville, MD, USA). Mutant KDR (GFP–S229A-KDR) was created with the QuickChange site-directed mutagenesis kit (Stratagene; La Jolla, CA, USA). All constructs were confirmed by DNA sequencing (Shanghai Shenggong Bio, China). Roscovitine was obtained from Sigma-Aldrich (R7772; St. Louis, MO, USA).

### Protein extraction and Western blot analysis

Frozen pituitary and pituitary adenoma tissues were harvested by washing with ice cold phosphate-buffered saline (PBS) three times and then scraped in ice-cold RIPA buffer (50 mM Tris, pH 7.5; 250 mM NaCl; 10 mM EDTA; 0.5% NP-40; 1 μg/mL leupeptin; 1 mM PMSF; and 4 mM NaF). The homogenates were sonicated three times for 6 s on ice, and centrifuged at 12,000 *g* for 5 min at 4°C to yield the total protein extract in the supernatants. Protein concentration was determined with a bicinchoninic acid assay (BCA) assay kit (Pierce). Protein samples (50 μg) were denatured, subjected to sodium dodecyl sulfate-polyacrylamide gel electrophoresis (SDS-PAGE) using 12% running gels, and transferred to nitrocellulose membranes. After blocking with 5% milk powder for 1 h at room temperature, the membranes were incubated with primary antibody, rabbit polyclonal anti-p35 antibody (1:100; Santa Cruz Biotechnology; sc-820) and GAPDH (1:5,000; Abcam; ab6276) overnight at 4°C.

### Wound-healing assay

After GH3 cells had grown to confluence in 35 mm culture plates, an artificial “wound” was created using a 10 μl pipette tip to scratch the cell monolayer. The wound area was inspected after 24 and 48 h using an inverted phase-contrast microscope with a digital camera. The wound healing speed was calculated as the percentage of the initial wound at different times until total wound closure.

### *In vitro* invasion assay

Assays were performed using Falcon cell culture inserts (8 μm pore size) in 24-well culture plates (BD Biosciences; Bedford, MA, USA) according to the vendor's instructions. Cell invasion was performed using Transwell chambers (8 μm pore size; Corning Costar Corp; Cambridge, MA, USA) with Matrigel (50 μg/mL; BD Biosciences). GH3 cells (10^5^ cells/well) in 0.5 ml of serum-free medium containing roscovitine at the indicated concentration were seeded onto Matrigel-coated membranes in the upper chambers and incubated at 37°C. The lower chambers contained the same amount of roscovitine in medium containing 10% FBS. After 24 h, cells that had invaded the lower chamber were fixed in 4% paraformaldehyde and stained with hematoxylin. Membranes were mounted on glass slides and observed using a phase-contrast microscope. Photographs were taken, and the number of cells in each of three randomly chosen high-power (200×) fields was counted. All experiments were performed three times.

### Statistical analysis

Results are presented as means±SD or medians and interquartile range (IQR), depending on data distribution. Proportions and frequencies were used for categorical variables. Comparisons between two groups were performed using Student's unpaired two-tailed t-test. Comparisons between three groups were performed using one-way ANOVA with Newman–Keuls test. Survival curves were calculated using the Kaplan-Meier algorithm and Log-rank (Mantel-Cox) test with GraphPad Prism 6.01. A P-value ≤0.05 was considered statistically significant.
